# Stop comparing spells: a defense of mechanistic reasoning in quantitative medicine

**DOI:** 10.3389/fdgth.2026.1878419

**Published:** 2026-07-27

**Authors:** Madhur Mangalam, Ken Kiyono

**Affiliations:** 1 Department of Biomechanics, University of Nebraska at Omaha, Omaha, NE, United States; 2 Graduate School of Engineering Science, University of Osaka, Toyonaka, Osaka, Japan

**Keywords:** clinical machine learning, interdisciplinary scientific literacy, mechanistic interpretability, model transparency, quantitative biomedicine

## Abstract

Mathematical methodology—spanning statistics, time series analysis, modeling, and AI-driven approaches—functions as a foundational *language* of biomedical research rather than a mere instrument, profoundly shaping the field by extracting structure from complex biological data. Yet as these methods grow more sophisticated, a quieter problem emerges: when the reasoning encoded in our methods is not made readable, scrutiny becomes harder—it grows more difficult to separate signal from artifact, mechanism from correlation, or insight from overfitting. The capacity for such distinctions is not lost to us; rather, the controls that data science has developed to enforce them are applied unevenly, and the expectation that quantitative methods remain legible to their users has eroded. We term this the “magicalization” of mathematics—the transformation of quantitative methods into oracular pronouncements accepted on faith or incantations rejected wholesale, rather than transparent arguments open to challenge. We treat this less as an accomplished verdict than as a tendency whose costs compound if current practice fails to correct it. This departs fundamentally from the vision of Poincaré and Wiener, who held that mathematics must function as a shared language sustaining dialogue between quantitative and domain experts. We readily concede that achieving operational understanding of mathematical constructs across disciplinary boundaries is inherently difficult; yet abandoning mutual intelligibility is neither scientifically sound nor methodologically neutral—it limits scrutiny and obstructs mechanistic understanding. We trace how magicalization manifests in clinical translation, black-box deployment, and research evaluation; identify its structural causes in complexity escalation, incentive misalignment, and disciplinary siloing; and argue that recovering mathematical interpretability serves as more than an academic luxury—it stands as a prerequisite for rigorous, mechanistically grounded biomedical science.

## 1 Introduction

Mathematical methodology—encompassing statistics, time series analysis, modeling, and AI-driven approaches—constitutes a foundational *language* of biomedical research rather than a mere instrumental toolkit. Through this language, researchers extract structure from complex biological data, articulate hypotheses with precision, and submit claims to communal scrutiny. Properly used, mathematics enables dialogue between quantitative and domain experts, each side literate enough to read and contest the other’s reasoning. This was the vision articulated by Norbert Wiener in his preface to *Cybernetics* [1], and before him by Henri Poincaré [2, 3]: interdisciplinary work succeeds when mathematics remains comprehensible across expertise boundaries. We invoke these figures not out of deference to tradition but because the value they defended is instrumental: the ability to read a method is what lets us interrogate its findings, recognize when a prediction is wrong, and detect when results reflect unanticipated structure in the data rather than the phenomenon of interest. It is that capacity, not fidelity to any historical ideal, that is worth preserving.

Achieving such comprehension is genuinely difficult—the authors readily concede partial mastery of the very methods we discuss—yet conceding difficulty differs sharply from abandoning the pursuit, and abandonment is what we diagnose here.

There is a recurring tendency to treat mathematical formulations as opaque operations whose internal logic escapes examination—either accepted on faith as oracular pronouncements, or rejected wholesale as suspicious incantations. This tendency is neither new nor unrecognized: the machine-learning and clinical-informatics communities have documented it repeatedly, from critiques of post-hoc explainability [4, 5] to analyses showing how data leakage and distribution shift yield models that excel in validation yet fail in deployment [6–9]. Our claim is not that researchers are blind to these failures, but that the correctives the field has developed remain unevenly adopted in biomedical practice, and that the expectation—that a method’s reasoning be legible to those who use it—has weakened.

We term this the “magicalization” of mathematics: the transformation of quantitative methods from transparent arguments into either oracles or incantations. When the reasoning encoded in our methods is not made readable, it becomes far harder to distinguish methods that capture genuine biological mechanisms from those that merely fit patterns in data—to separate signal from artifact, insight from overfitting, or understanding from correlation.

This matters because, to the extent a research enterprise comes to rest on unreadable methods, it grows fragile in predictable ways. It adapts poorly when contexts shift, is slow to notice when its instruments begin to fail, and struggles to distinguish durable findings from sophisticated curve-fitting. This is a trajectory rather than a verdict, and it remains far from inevitable. It results from specific structural choices—in how we train researchers, evaluate research, and design our methods—and different choices remain possible.

This essay examines how magicalization occurs, why it persists, and how recovering mathematical interpretability could restore the Wiener–Poincaré ideal.

*Our argument is ultimately a defense of mechanistic reasoning*. The capacity to distinguish mechanism from correlation depends on being able to read the mathematical methods we employ. When mathematics becomes opaque—accepted as oracle or rejected as incantation—we lose the ability to evaluate whether our methods capture biological processes or merely statistical patterns. Interpretability serves as a means rather than an end; it is the prerequisite for mechanistic science. Without it, we are left with empirical validation alone: methods that predict well but teach nothing, that work until they fail, whose reasoning eludes reading and whose failures resist diagnosis.

## 2 Three dimensions of mathematical transparency

Before proceeding, we must distinguish three related but distinct concepts that are often conflated in discussions of quantitative methods.

**Interpretability** concerns the *transparency of the mathematical formulation itself*. Critically, interpretability extends beyond a technical property of models—it functions as a social and epistemic requirement that enables users to evaluate, contest, and trust the conclusions drawn from quantitative methods [10]. A linear regression model is interpretable: we can examine the coefficients, understand what each variable contributes, and follow the logic connecting inputs to outputs. A deep neural network with millions of parameters lacks interpretability in this sense: even though the computation is precisely defined, the reasoning it encodes resists reading by human observers.

**Mechanistic validity** refers to whether a method captures genuine biological mechanisms or merely statistical patterns. A model that uses heart rate variability metrics grounded in autonomic physiology is mechanistically valid. A model that achieves the same predictive performance by exploiting correlations with hospital coding practices fails this standard—even when both models are equally interpretable mathematically.

**Interrogability** refers to whether a method’s behavior can be systematically probed and tested, even when the method itself resists full interpretation. A black-box model becomes interrogable when we can perform sensitivity analyses, counterfactual testing, or local explanation techniques to understand how it behaves under different conditions. Interrogability is about *whether we can critically examine the method’s reasoning indirectly, when direct reading is impossible*.

These three dimensions are independent. A method can be:

- Interpretable but mechanistically invalid (a simple regression model using spurious predictors)
- Mechanistically valid but resistant to full interpretation (a physiologically grounded simulation with complex nonlinear dynamics)
- Interpretable and mechanistically valid but poorly interrogated (a good model deployed without testing its failure modes)
- Opaque yet interrogable (a neural network subjected to rigorous behavioral analysis)

Our argument concerns primarily *interpretability*—the capacity to read mathematical methods as arguments rather than merely accepting them as authoritative oracular outputs. However, interpretability serves a deeper scientific purpose: it enables evaluation of mechanistic validity and facilitates meaningful interrogation of a method’s assumptions, scope, and limitations. When we can read the mathematics, we are better positioned to assess whether it captures something biologically real, whether its operations map plausibly onto underlying mechanisms, and where or why it is likely to break down. When reading becomes impossible, these critical functions grow far more difficult, though some remain partially achievable.

The magicalization we diagnose is the abandonment of interpretability as a scientific standard. This matters less because all methods must be simple—complexity is often necessary—and more because *unreadable methods resist critical evaluation*. They elude challenge on mechanistic grounds. They escape effective interrogation because we lack the questions to ask. They must be accepted on faith or rejected wholesale, with no middle ground for reasoned critique.

The Wiener–Poincaré ideal was that mathematics should function as a shared language enabling dialogue between quantitative and domain experts. This requires interpretability: both parties must be able to *read* the argument being made. Mechanistic validity and interrogability matter deeply, yet both presuppose a foundation of interpretability. Assessing whether a method captures real mechanisms requires understanding what the method is doing. Interrogating a black box effectively requires the conceptual vocabulary to frame meaningful questions.

In what follows, we focus on interpretability while recognizing its relationship to these other dimensions of transparency. The goal extends beyond mathematical simplicity for its own sake, emphasizing instead mathematical *readability* as a prerequisite for scientific accountability.

## 3 Historical context: the ideal of mathematical literacy

To understand what has been lost, we must first establish what once existed—or at least what was envisioned. Let us begin with an honest admission: mathematics is hard. The authors of this essay readily confess that our own grasp of the mathematical machinery we rely upon remains partial, and we suspect most readers will recognize this experience. Every working scientist accumulates methods whose inner workings exceed the time and attention any single career affords. Physicists invoke functional analysis, engineers apply spectral methods, and mathematicians themselves routinely employ results from neighboring subfields whose proofs they have absorbed only in outline. This shared partiality reflects an unavoidable feature of laboring at the frontier of mathematically intensive disciplines, where the depth of available technique always outruns any individual’s mastery. Friction between mathematics and the biological sciences has therefore always been present, just as it persists in physics and engineering. Yet at critical moments, influential thinkers articulated a clear and more modest ideal: mathematics should function as a *language* that enables precise communication and mutual critique across disciplines—demanding from all parties enough shared comprehension to permit critical dialogue, while leaving room for the inevitable gaps in any one practitioner’s expertise. It is this ideal, rather than an unattainable standard of universal rigor, that we argue has been abandoned. No one captured this vision more lucidly than Henri Poincaré.

### 3.1 Poincaré’s vision of complementary expertise

Henri Poincaré, writing at the turn of the 20th century, addressed the relationship between mathematics and physics in terms that remain relevant to contemporary biomedicine. In *Science and Hypothesis* [2, 3], Poincaré argued against rigid disciplinary boundaries. The physicist, he insisted, need not prove theorems—but must understand mathematical reasoning well enough to judge whether a derivation is logically sound and whether its conclusions map meaningfully onto physical reality. Conversely, the mathematician working on physical problems cannot treat physics as a mere source of interesting equations; they must grasp the physical principles well enough to recognize when a mathematical result, though formally correct, is physically absurd or irrelevant.

Poincaré’s framework rests on a crucial insight: *the validity of a mathematical statement and the meaningfulness of its application are distinct questions*. A theorem may be impeccably proven yet utterly useless for a given physical problem. Conversely, an approximate or heuristic argument may capture essential physics despite lacking formal rigor. This distinction—between formal correctness and scientific utility—is precisely what gets lost when mathematics is treated as magic. Researchers can no longer navigate between these two dimensions of evaluation. They are left either accepting results on authority or rejecting them out of suspicion, unable to critically assess both logical validity *and* interpretative relevance.

### 3.2 Wiener’s cybernetic ideal

Norbert Wiener, writing nearly half a century later, extended this vision explicitly to the life sciences. The preface to *Cybernetics* [1] is remarkably prescient in anticipating the challenges of interdisciplinary quantitative work. Wiener had observed firsthand the friction between mathematicians, engineers, and physiologists during wartime research collaborations. His proposed solution rejected siloed expertise in favor of bidirectional literacy.

The mathematician, Wiener wrote, must be able to “understand” a physiological experiment—less in the sense of personally conducting it than in the sense of grasping its logic, recognizing its limitations, and proposing refinements or alternatives. Similarly, the physiologist must be able to “follow” mathematical reasoning—meaning more than mere acceptance of conclusions, extending to understanding the chain of inference connecting premises to results, and thereby judging whether those results bear on the biological question at hand.

Wiener’s emphasis on “understanding” and “following” is critical. He stopped short of advocating that physiologists become mathematicians, or vice versa. Rather, he insisted that each discipline develop sufficient literacy in the other’s language to engage in genuine *dialogue*. This dialogue is what enables the iterative refinement essential to good science: the mathematician proposes a model; the physiologist identifies where it diverges from biological reality; the mathematician revises the formulation; and so on. Without mutual intelligibility, this loop breaks down.

Importantly, Wiener recognized that this ideal required institutional support. Individual researchers possessing broad training proved insufficient; the *culture* of research had to value and reward interdisciplinary fluency.

### 3.3 What has changed

What has changed since Wiener’s time is not the ideal, but the practice. The expectation that mathematical methods be interpretable to their intended audience has weakened. The middle ground—where quantitative and domain expertise meet in genuine dialogue—has narrowed rather than vanished, but it has narrowed enough to be worth actively defending.

Several structural shifts can be identified. The sheer *volume* and *complexity* of mathematical methods in biomedicine have increased exponentially. In Wiener’s era, a physiologist engaging with mathematical models might encounter differential equations or compartmental models. The growth of computationally sophisticated methods in biomedicine over the past decade is well documented [11–13]. Today, a single study might combine spectral analysis, nonlinear dynamics, information theory, Bayesian inference, and machine learning, each drawing on a distinct body of specialized training.

*Publication norms* have shifted in ways that discourage interpretability. Journals increasingly prioritize novelty, statistical significance, and performance metrics over mechanistic insight [14–16].

*Regulatory and clinical frameworks* have adapted to complexity by relying on black-box validation rather than mechanistic transparency. A diagnostic algorithm need not be interpretable to receive FDA clearance; it must simply demonstrate acceptable performance, and analyses of cleared devices show that many are authorized on retrospective, single-site performance alone, without prospective or multi-site evaluation [17–19].

Finally, the *cultural expectation* of mathematical literacy has declined. It is increasingly acceptable for a physician-scientist to delegate all quantitative work to collaborators, and for a quantitative researcher to publish biomedical applications without deep engagement with the relevant physiology [20, 21]. The middle ground—researchers who are fluent enough in both languages to bridge them—has become rare and undervalued.

## 4 How magicalization manifests

The consequences of treating mathematics as magic are not abstract. They manifest in concrete, observable ways across the research lifecycle.

### 4.1 Clinical translation: the institutionalization of opacity

The magicalization of mathematics appears most visibly in clinical practice, where it has become embedded in regulatory frameworks, clinical guidelines, and medical devices.

Consider QTc correction formulas—a seemingly mundane example that illustrates the phenomenon clearly. The QT interval on an ECG shortens as heart rate increases. To assess arrhythmia risk, we need a rate-corrected QT (QTc). Multiple formulas exist: Bazett’s [22], Fridericia’s [23], Framingham [24]. These give *different answers*. Bazett’s formula systematically overcorrects at high heart rates and undercorrects at low heart rates. The choice of formula can change clinical decisions—whether to label a QT as prolonged, whether to withhold a medication. Multiple studies have documented the clinical significance of formula choice [25, 26].

Yet in practice, QTc is often treated as a single objective number delivered by the ECG machine. The clinician may remain unaware of which formula is being used, much less how it works or where it fails. The number is simply accepted: the machine says 420 ms, therefore we treat it as prolonged.

This is revealing. The correction formula is straightforward—a simple algebraic transformation that any medical student could grasp in minutes. Yet such understanding is rarely expected. The formula is embedded in infrastructure, and its output is accepted on faith.

This example reveals an important distinction. The QTc formulas are highly *interpretable*—any medical student can read Bazett’s formula ($\text{QTc}=\text{QT}/\sqrt{\text{RR}}$) and understand what mathematical operation is being performed. Yet their *mechanistic validity* remains contested: which formula best captures the true physiological relationship between heart rate and ventricular repolarization? Bazett’s overcorrects at high rates less because it is mathematically wrong than because the assumed square-root relationship may diverge from the underlying electrophysiology [25]. Here we have transparency without mechanistic certainty. We can read the argument, debate its biological validity, and recognize its limitations. This is precisely the kind of critical engagement that magicalization prevents. When formulas are embedded in devices and outputs accepted without question, even interpretable methods become oracular—less because they are opaque than because understanding has ceased to be expected.

The stakes are relatively low for QTc because the formulas are simple and well-studied. The stakes become much higher when we consider modern algorithmic medicine: risk calculators that determine who receives statins, scoring systems that ration ICU beds, machine learning models that guide chemotherapy decisions. These are anything but simple formulas. They are complex computational systems, often proprietary and rarely transparent. Yet they receive the same faith-based acceptance: the algorithm says X, therefore X is true.

### 4.2 The black-box problem

The problem becomes acute with machine learning in clinical decision support.

A neural network trained to predict sepsis from electronic health records might achieve impressive performance: high sensitivity, reasonable specificity, good calibration. It is deployed clinically. Clinicians receive real-time risk scores: “Patient in Bed 4 has 73% probability of sepsis in next 6 h.” Such systems have been widely deployed [27, 28], yet external validation studies reveal concerning performance degradation [9].

What does this number mean?

The honest answer is: we lack precise knowledge. The model is a black box. It ingests hundreds of variables—vital signs, lab values, medications, demographics—and transforms them through millions of learned parameters. The mapping is mathematically well-defined but interpretable only in the trivial sense that we can trace the computation. We struggle to explain, in biological terms, *why* this particular patient received this particular risk score.

Suppose the algorithm flags a patient who looks clinically stable: normal vital signs, no overt signs of infection. Should the clinician trust the model and escalate care anyway? Or trust their clinical judgment and wait?

This decision hinges on *why* the model flagged the patient. Is it detecting subtle patterns in vital sign trajectories that herald decompensation? Or has it latched onto confounders in the training data—a problem documented in algorithmic bias research [29]—perhaps this patient’s demographic profile or medication list resembles septic patients, even though the underlying physiology differs?

This is fundamentally a failure across all three dimensions of transparency. The model lacks *interpretability*: we cannot read its reasoning in any meaningful sense. Its *mechanistic validity* remains unknown: we cannot tell whether it captures genuine physiological precursors to sepsis or has learned spurious correlations. And crucially, it has gone inadequately *interrogated*: we have failed to systematically test which features drive predictions, whether those features are physiologically plausible, or how the model behaves when populations shift.

Without interpretability, the clinician fails to distinguish these scenarios. They must either follow the algorithm blindly or ignore it entirely. Neither qualifies as science. But notice: even when full interpretability lies beyond reach, interrogation remains possible. We could perform sensitivity analyses to identify which variables matter most. We could test counterfactuals: if this patient’s lactate were lower, would the prediction change? We could examine failure cases: when the model is wrong, what characterizes those patients? Such interrogation would stop short of rendering the black box transparent, yet it would make it *contestable*—subject to critical challenge rather than blind faith.

The tension between accuracy and explainability in clinical AI has been extensively debated [4, 5]. But the debate often conflates interpretability with interrogability, assuming that incomplete reading forces blind trust in outputs. This claim fails. The alternative to full transparency lies in systematic interrogation rather than blind acceptance.

The standard response is that interpretability is optional if the model works. But “works” is subtle. Validation studies measure average performance across populations. They offer no guarantee the model works for *this patient, now*. Clinical medicine centers on particular cases unfolding within specific contexts, rather than on population averages.

Moreover, “working” itself requires interpretation. Suppose the sepsis model has 80% sensitivity and 85% specificity. Is that good? It depends on what the model is actually detecting. If it captures early physiological instability—subtle trends that precede overt sepsis—then yes, this performance is valuable. But if it mostly picks up on coding artifacts or measurement errors, then no, it is overfitting to the training distribution and will fail when deployed in new contexts.

Validation metrics alone fail to reveal the difference. You must interrogate the model’s behavior. Yet when the culture accepts black boxes as legitimate scientific outputs, this interrogation never happens. We deploy first, ask questions later—if at all.

### 4.3 Research evaluation: when understanding becomes optional

The magicalization of mathematics also appears in how research is evaluated. This manifests most clearly in peer review, where the structural incentives of publication can unintentionally reinforce a view of mathematics as oracle rather than argument.

Consider a prototypical scenario in biosignal analysis. A manuscript computes multiple indices from physiological time series—perhaps measures of heart rate variability in patients with autonomic dysfunction. Each index is grounded in a different mathematical framework: one captures temporal variability, another spectral content, a third nonlinear complexity. The paper explains what each mathematical operation means biologically and demonstrates which aspects of the signal carry clinical information.

A review returns with a familiar request: “The authors should compare which index performs best. A table ranking by AUC would strengthen the paper.”

This comment appears reasonable. Empirical comparison is legitimate practice. But notice the questions left unasked: What does this index measure? What physiological process corresponds to the mathematical quantity being computed? If Index A outperforms Index B, is that because A captures something B misses, or because A overfit to this dataset? What would it mean, mechanistically, for A to be “better”?

These questions probe understanding. They treat mathematics as a language that encodes claims about biology—claims that can be insightful or vacuous, meaningful or merely correlational. But in contemporary practice, such questions have become optional. What matters is the number: which method wins the comparison.

To see why this is problematic, suppose we are analyzing heart rate variability with:

- SDNN (standard deviation of NN intervals): total variability across all timescales
- RMSSD (root mean square of successive differences): beat-to-beat variability, reflecting parasympathetic modulation
- LF/HF ratio (spectral power ratio): often interpreted as sympathovagal balance, though contested
- Sample entropy: irregularity of the time series, taken to reflect control complexity

These indices are well-established in the literature [30–32], though their physiological interpretation remains contested [33].

Which is “best”? The question makes no sense without specifying what we mean. These indices are highly *interpretable*—we can read the mathematical operations and understand what each computes. Their *mechanistic validity*, however, is contested. Does RMSSD truly isolate parasympathetic modulation, or does it capture something more complex? Does LF/HF ratio reflect sympathovagal balance, as commonly claimed, or is this interpretation oversimplified [33]? These are genuine scientific questions about whether the mathematics maps onto physiology in the ways we assume.

If RMSSD predicts mortality better than SDNN in a cohort, that fails to establish RMSSD as “better.” It establishes instead that *in this population, rapid parasympathetic fluctuations (or whatever RMSSD actually measures) were more prognostically relevant than total variability*. That is a scientific finding suggesting a mechanistic hypothesis worth investigating. But framing it as “RMSSD wins” obscures the biology. It treats these indices as competing spells rather than as different mathematical operationalizations of different physiological constructs, each with its own mechanistic claims and limitations.

This is where interpretability enables mechanistic reasoning. Because we can read what SDNN and RMSSD measure—what mathematical operation each performs—we can ask: what physiological process corresponds to this operation? Is that correspondence valid? When would it break down? Without interpretability, these questions become impossible. The methods become incantations: RMSSD worked, SDNN failed, the reason eludes us, moving on.

When equations are opaque—when these are just names attached to software outputs—all that remains is performance comparison. The methods become incantations: RMSSD worked, SDNN failed, the reason eludes us, moving on.

The consequences accumulate. If reviewers demand only performance comparisons, authors will optimize for that metric. They will test multiple indices, report the top performer, and omit mechanistic interpretation. Over time, the literature fills with methods that are empirically validated yet conceptually opaque. No coherent understanding emerges, because understanding was never the objective in the first place.

In some cases, this dynamic suggests that mathematical reasoning itself has become a kind of magic for the reviewers—something to be evaluated by its results rather than understood as a line of argument. This critique targets structural dynamics rather than individuals; it reflects how publication pressures can inadvertently encourage treating mathematics as an oracle rather than as a language.

## 5 Structural causes

Why has magicalization become so pervasive? Several structural factors reinforce it.

### 5.1 The complexity ratchet

That biomedical methods have grown more complex is, by itself, no cause for alarm—and not the heart of the problem. Complexity need entail no opacity. What matters is not how much computation a method performs but whether its reasoning remains legible, and on that axis methods divide sharply. The crucial distinction is between *compositional* methods, which break down into understandable steps, and *holistic* methods, whose outputs emerge from the system as a whole. A genome-wide association study involves enormous statistical complexity, yet its core logic remains conceptually accessible. A deep neural network, by contrast, distributes its computation across millions of parameters with no clean intermediate representations to point to [34, 35].

This matters because compositional complexity can be taught: explain the pieces and the whole becomes comprehensible. Holistic complexity resists such teaching. As holistic methods proliferate, the expectation of interpretability declines, and the standard shifts from “explain how it works” to “demonstrate that it works.” This is the complexity ratchet: each generation of methods is less interpretable than the last, and each generation normalizes lower standards of transparency.

### 5.2 Incentive misalignment

Academic incentives reward novelty and performance metrics over interpretability. A paper showing that Method X outperforms existing methods is publishable; a paper explaining *why* Method X works—what it captures, what it assumes, where it will fail—is often dismissed as speculative or beyond scope. We have encountered this pattern directly in the review process, where requests for performance comparisons routinely supersede inquiries into mechanistic interpretation. Journals favor clear, quantifiable results: AUC values, p-values, effect sizes. Mechanistic interpretation is harder to formalize, harder to referee, and harder to market [36].

Funding follows the same logic. Grant applications promising to “develop a machine learning model to predict X” are legible to review panels, while applications promising to “understand what physiological features predict X” sound vague. The former gets funded; the latter struggles.

Smaldino and McElreath [16] have modeled this dynamic formally, showing how scientific incentive structures select for methods that are publishable over methods that are true or interpretable. When success depends on novelty and performance metrics rather than mechanistic insight, researchers rationally optimize for what is rewarded. The problem lies in structure rather than individual venality: the system systematically disincentivizes the very qualities—interpretability, mechanistic grounding, cautious interpretation—that make scientific knowledge reliable.

### 5.3 The clinical imperative

Medicine faces urgent practical needs. If a black-box algorithm can predict sepsis before it becomes overt, should we refuse to use it merely because its workings remain obscure? The pragmatic answer is often no—and yet this framing is too simple.

Interpretability is *essential for safety*. Black boxes fail silently and in ways that correlate with biases in their training data; an algorithm trained on one population may fail catastrophically when deployed in another, with the failure invisible until harm occurs. Moreover, the urgency argument assumes a false choice between interpretable and effective. Many problems admit interpretable solutions, but such solutions are harder to develop, demanding deeper engagement with physiology and more careful mathematical modeling. Under time and funding pressure, researchers default to black boxes because they train faster and validate more easily.

The clinical imperative thus becomes justification for intellectual shortcuts. We accept opacity because we are in a hurry, and because shifting standards have made opacity acceptable. The long-term cost is clinical infrastructure built on methods we lack the means to repair when they fail.

### 5.4 Disciplinary siloing

Perhaps the deepest cause is cultural: the normalization of disciplinary boundaries that absolve each side of responsibility for understanding the other. A clinician-scientist may disclaim quantitative work entirely and delegate it to collaborators; a quantitative researcher may publish biomedical applications while relying on domain experts for superficial validation.

This division of labor seems efficient but undermines the Wiener ideal. The clinician who lacks mathematical understanding cannot critically evaluate whether quantitative conclusions are trustworthy; the mathematician who lacks physiological understanding cannot judge whether their results are biologically meaningful. Each side produces half an argument, and responsibility for the whole falls to no one [37].

The dynamic is self-reinforcing. As fewer researchers occupy the middle ground, training programs cease to cultivate the bridging skills it requires. Medical students receive less mathematical training; mathematicians engage less deeply with biology. Each generation inherits a landscape in which interdisciplinary dialogue is no longer expected, and the middle ground fades from the culture altogether.

### 5.5 Documented consequences of opacity

These harms are far from speculative. When researchers cannot read the reasoning encoded in their methods, predictable failures follow.

Obermeyer et al. [29] found that a widely deployed risk algorithm systematically underestimated illness severity in Black patients because it optimized health care costs as a proxy for needs. The conceptual error remained invisible to users who treated the algorithm’s output as objective measurement rather than the conclusion of a specific—and questionable—mathematical argument. Wong et al. [9] found that most published COVID-19 prediction models failed when tested outside their training contexts, having learned local patterns rather than portable mechanisms. Zech et al. [38] showed that neural networks trained to detect pneumonia from chest X-rays were actually identifying which hospital acquired the image, exploiting institutional artifacts rather than disease features.

These cases share a structure: methods treated as oracles rather than arguments fail in ways that would have been predictable had the reasoning been readable. The problem extends beyond machine learning. Vandenberk et al. [26] demonstrated that QTc formula choice affects clinical classification of arrhythmia risk, yet clinicians routinely accept QTc values without knowing which formula was used or where it fails. Ioannidis [15] and Begley and Ellis [39] documented widespread irreproducibility driven partly by analytical flexibility that remains invisible when methods are treated as standardized procedures rather than chains of reasoning requiring justification.

When mathematical operations become detached from readable arguments, we lose the ability to distinguish methods that capture genuine mechanisms from those that merely fit data. The consequences are measurable: biased algorithms deployed at scale, models that fail when contexts shift, research findings that resist replication.

## 6 Why this matters

### 6.1 The legitimate case for complexity

The argument for mathematical interpretability must confront its strongest challenge: some problems may genuinely require methods whose reasoning cannot be made fully transparent, yet whose use is justified by superior outcomes.

Consider a scenario: two models predict imminent cardiac arrest in ICU patients. Model A uses five physiological variables with clear mechanistic rationale (heart rate variability, respiratory rate trend, blood pressure trajectory, lactate, temperature), achieving 75% sensitivity. Model B uses hundreds of variables in learned combinations that resist full interpretation, achieving 85% sensitivity. The 10-point improvement could save lives. Should we refuse Model B on principle?

This is far from a strawman. Caruana et al. [40] encountered this dilemma in pneumonia risk prediction and argued for interpretable models despite performance trade-offs. Rudin [5] has forcefully challenged the assumption that high-stakes decisions require black boxes. Yet the tension remains: if Method B genuinely saves more lives, and we have exhausted efforts to make interpretable methods competitive, what then?

We offer three responses.

First, the framing itself deserves scrutiny. The supposed trade-off between interpretability and performance often reflects *insufficient investment in interpretable alternatives* rather than fundamental limitations. Black boxes are faster to develop because they automate the hard work of identifying relevant features and relationships. Building interpretable models requires deeper engagement with domain knowledge, more careful feature engineering, more iterative refinement between quantitative and clinical experts. Under publication and funding pressure, researchers default to the path of least resistance. When Caruana et al. invested the effort, they built interpretable models that matched black-box performance.

Second, even accepting the trade-off, the question concerns less whether to use complex methods than *how*. A complex model subjected to systematic interrogation—sensitivity analysis, counterfactual testing, error pattern analysis, subgroup validation—differs qualitatively from one accepted on faith. The former remains an *argument* whose reasoning can be probed, even when full reading lies out of reach. The latter becomes an *oracle*. The distinction matters because interrogation enables us to detect failures before they cause harm, to understand when the method will break down, to maintain critical distance rather than blind trust.

Third, urgency has limits as justification. For established clinical problems, we have decades of physiological research identifying relevant features. That a black box slightly outperforms a mechanistic model often means we have yet to invest sufficient effort in understanding which physiological signals matter and how to measure them properly. The pressure to deploy quickly creates a cycle: we accept black boxes for expediency, which reduces incentive to develop interpretable alternatives, which normalizes black boxes further. This is the complexity ratchet again, but now defended as clinical necessity.

There may exist problems genuinely intractable to interpretable methods—where causal structures are too complex, interactions too numerous, mechanisms too poorly understood. But demonstrating genuine intractability requires systematic attempts to build interpretable solutions, attempts rarely made before defaulting to black boxes. The claim of intractability often serves as premature justification for opacity.

Moreover, even for intractable problems, *partial* interpretability suffices for scientific accountability. Global understanding of a model may elude us, but we can understand its behavior locally: for this patient, which features drove the prediction? Are those features clinically plausible? Do they suggest a mechanistic hypothesis we can test? Such local interrogation [41, 42] preserves the possibility of dialogue between quantitative and domain experts—the Wiener ideal—even when complete transparency is impossible.

The legitimate case for complex methods, then, is narrow: genuinely intractable problems, where interpretable approaches have been systematically attempted and failed, where complex methods are rigorously interrogated rather than blindly trusted, and where the performance gain is substantial enough to justify the epistemic cost of reduced transparency. This describes a small fraction of current practice.

What we oppose is something other than occasional, carefully justified use of opaque methods. We oppose the *normalization* of opacity as standard practice. We oppose reviewers who demand performance comparisons while ignoring mechanistic questions. We oppose algorithms deployed without interrogation. We oppose methods published and trusted despite being unreadable to their intended users. We oppose the cultural shift that treats “it works” as sufficient justification, without asking what “works” means, how it works, or when it will fail.

This is magicalization: the abandonment of mathematical reasoning as readable argument, subject to critical challenge by those who use it. Even when complexity proves necessary, this abandonment never does.

### 6.2 Beyond performance metrics: what “working” actually means

The magicalization of mathematics extends beyond academic concern, systematically undermining the reliability, generalizability, and long-term cumulative progress of biomedical research by obscuring how conclusions are reached.

Consider what “working” actually means. A method validated in one population may fail systematically in another—less from random variation than from a difference in underlying physiology. Without understanding what the method measures, these failures elude anticipation. Each new context requires re-validation from scratch, an infinite regress of empiricism that mistakes correlation for knowledge.

Worse, opacity prevents refinement. When a method fails, an interpretable approach can be diagnosed and improved: confounders identified, assumptions adjusted, scope clarified. An opaque method can only be discarded and replaced. We trade cumulative progress for perpetual restarts.

Most fundamentally, we risk mistaking the goal of science. Prediction and understanding differ fundamentally. A model that predicts mortality without explaining *why*—which physiological features matter, how they interact, and when the relationship breaks down—fails to constitute scientific knowledge; it remains a correlation presented as insight, one that may appear effective today yet falter as populations shift, measurement protocols change, or novel conditions arise. And when it fails, the reason will elude us, because we never understood how it worked.

This is the difference between science and sophisticated curve-fitting. Science produces portable knowledge: understanding that adapts to new contexts because it captures genuine mechanisms. Curve-fitting produces brittle instruments: they work until they fail, and their failures are silent and catastrophic. Clinical deployment of interpretable models has demonstrated that transparency need cost no performance [40]. Indeed, the application of fractal and multiscale analysis to physiological signals exemplifies how mechanistically grounded methods can provide both predictive power and interpretability [43–45].

The Wiener–Poincaré vision was never about mathematical purism. It was about building knowledge that lasts—methods whose reasoning we can read, whose assumptions we can challenge, whose failures we can diagnose. An opaque method that predicts well is useful in the way a lucky guess is useful: it helps once, teaches nothing, and leaves us no better prepared for the next problem.

### 6.3 The three dimensions in practice: what we gain and lose

The framework of interpretability, mechanistic validity, and interrogability clarifies what is at stake in different scenarios and what kinds of scientific engagement remain possible. These dimensions matter because they determine whether methods can function as arguments subject to critical challenge—a fundamentally social and communicative requirement [10].

**High interpretability, contested mechanistic validity** (e.g., QTc formulas, HRV indices): Here we can read the mathematics clearly, but disagree about whether it captures what we think it captures. This is normal science. We can debate the mechanistic assumptions, design experiments to test competing interpretations, and refine our understanding iteratively. The mathematics functions as Wiener envisioned: a shared language enabling productive disagreement.

**Low interpretability, high interrogability** (e.g., well-studied neural networks with extensive sensitivity analysis): Here full reading of the reasoning lies out of reach, yet we can probe its behavior systematically. We can identify which features matter, test whether the model’s dependencies align with physiological knowledge, and detect failure modes before deployment. This falls short of ideal—we still lack the transparency Poincaré advocated—yet it preserves the possibility of critical challenge. The method remains contestable even when full readability lies out of reach.

**Low interpretability, low interrogability** (e.g., proprietary algorithms deployed without behavioral analysis): This is magicalization in its pure form. The reasoning eludes reading, and the behavior has gone uninterrogated. The method becomes oracular: it produces outputs we must either accept on faith or reject wholesale. No middle ground exists for reasoned critique. This is the condition we must refuse to normalize.

The point is that methods need achieve no perfect score on all three dimensions; what ultimately matters is that *some meaningful form of transparency must be preserved* to enable critical evaluation. When interpretability lies out of reach, interrogability becomes essential. When mechanistic validity remains uncertain, interpretability enables debate. When all three dimensions collapse—when reading the method, interrogating its behavior, and validating its mechanistic claims all become impossible—we have left the domain of science entirely.

Current practice increasingly accepts this total collapse as legitimate, provided the method “works” in validation studies. This is what we oppose. Our target is neither complexity, nor sophistication, nor mathematical abstraction, but the abandonment of any requirement that methods remain open to critical challenge.

## 7 Restoring the language

Reversing the magicalization of mathematics will require changes at multiple levels: how we train researchers, how we evaluate research, and what we demand from our methods.

### 7.1 Reviving mathematical literacy

Medical training must include more than statistics taught as cookbook procedures; it must include genuine mathematical reasoning: how to read an equation, how to assess whether a mathematical claim follows logically from its premises, how to judge whether a quantitative method is appropriate for a given question. The current state falls far short of this ideal. Most internal medicine residents struggle to correctly interpret basic statistical results in published literature, a failure that reflects systematic curricular neglect rather than individual deficiency [20, 21].

This implies no aim of turning physicians into mathematicians. It means providing the literacy Wiener described: enough fluency to follow an argument and recognize when it breaks down. A medical student who understands basic calculus, linear algebra, and probability theory has the instruments to engage critically with most quantitative methods in biomedicine. The limiting factor lies in curricular priorities rather than intellectual capacity.

Conversely, training for quantitative researchers must include substantive engagement with physiology and clinical medicine. A statistician or engineer working on biosignals should understand autonomic regulation, cardiac electrophysiology, or respiratory mechanics at more than a superficial level. They need the domain knowledge to recognize when a mathematical result, though formally correct, is biologically implausible.

This is demanding. It requires researchers to invest years developing competence in a second field. But it remains essential if we want to reverse magicalization. The middle ground Wiener described arises through no spontaneous process—it must be deliberately cultivated through training and institutional support.

### 7.2 Changing evaluation standards

Journals must stop accepting black-box validation as sufficient. A paper that presents a new method should be required to explain:

1. What does this method measure? What mathematical operation is being performed, and what does that operation correspond to conceptually?
2. Why would we expect this quantity to contain information about the biological process of interest? What is the mechanistic hypothesis linking the mathematical representation to the physiology?
3. How does this method relate to existing methods? Is it measuring the same thing differently, or measuring something orthogonal? What assumptions does it make that existing methods do not?
4. Where will this method fail? What kinds of signals, measurement conditions, or physiological states will produce misleading results? How can users detect these failure modes?

These points warrant elevation from optional discussion to core scientific requirements. A paper that fails to answer them should remain unpublished, regardless of performance metrics, as performance alone falls short of replacing clarity about what a method measures and how it operates.

Reviewers must be trained—and incentivized—to ask these questions. This means recruiting reviewers with genuine interdisciplinary expertise: people who can read both the mathematics and the biology critically. It also means recognizing that “the method works,” on its own, falls short of validating the method as sound.

These are not new demands. Reporting frameworks such as TRIPOD+AI, MI-CLAIM, and CONSORT-AI/SPIRIT-AI already codify much of what authors should disclose about model development, validation, and failure modes [46–48]. The gap lies less in the absence of standards than in their uneven enforcement: reviewers and editors who insist on demonstrated stability—out-of-distribution testing, subgroup performance, and explicit failure analysis—turn magicalization from a default into a choice an author must actively defend.

### 7.3 Demanding interpretability from methods

Few methods can be fully interpretable. Some problems genuinely require complex, opaque computations. But even then, we can demand *interrogability*: the ability to probe the method’s behavior systematically.

For machine learning models, this means:

- Sensitivity analysis: how do predictions change when inputs vary? Which variables matter most?
- Counterfactual analysis: what would need to change for the prediction to flip? Are those changes physiologically plausible?
- Error analysis: when the model fails, what characterizes the failure cases? Is there a systematic pattern suggesting what the model is actually learning?

The explainable AI community has developed numerous such techniques [49, 50].

These techniques stop short of rendering a black box transparent, yet they make it *contestable*. They allow domain experts to challenge the model’s reasoning indirectly, by examining whether its behavior aligns with physiological knowledge.

Critically, this interrogation should happen *before* deployment rather than after. A model deserves no validation merely from achieving good AUC on held-out data. It should be interrogated for failure modes, tested for robustness to distribution shift, and evaluated for alignment with mechanistic expectations. Only after passing these tests should it be deemed safe for clinical use.

### 7.4 Valuing understanding over prediction

Most fundamentally, we must resist the cultural shift that treats prediction as the sole goal of biomedical research.

Prediction is important. But it differs from understanding, and in the long run, understanding is what enables progress. A predictive model that works today may fail tomorrow when the population changes, when measurement protocols shift, when a new pathogen emerges. Lacking insight into *why* the model worked, we lose the ability to adapt it. We are left retraining from scratch, in an endless cycle of empirical fitting.

In contrast, mechanistic understanding is portable. If you understand the physiology well enough to design methods that capture the relevant features explicitly, those methods adapt as contexts change. They may forgo the absolute highest predictive performance in any given dataset—overfit models can always win on that metric—yet they remain useful across a wider range of conditions. They fail gracefully and predictably, rather than silently and catastrophically.

This is the trade-off we are currently getting wrong. We are optimizing for short-term performance at the cost of long-term robustness. We are building a biomedical research enterprise on methods we fail to understand and lack the means to maintain. Despite calls for more interpretable approaches in high-stakes medical decisions [11–13], the trend toward opacity continues.

## 8 Conclusion

The magicalization of mathematics in medicine represents an epistemological crisis: the abandonment of the ideal that quantitative methods should be readable, interpretable, and subject to critical challenge by their intended audience.

When equations become incantations—when they are treated as oracles whose pronouncements must be accepted on faith, or as suspicious rituals to be rejected wholesale—we lose the capacity for genuine interdisciplinary science. We are left with parallel monologues: mathematical specialists producing outputs that domain experts struggle to critique, and domain experts raising objections that mathematical specialists struggle to parse. The middle ground, where actual collaboration occurs, disappears.

This matters because biomedical research is no spectator sport. Having someone, somewhere, who understands the methods while everyone else accepts the results on authority falls short of what science requires. Every researcher engaging with quantitative methods must develop sufficient literacy to read the mathematics critically—to judge whether the claims being made are logically sound and whether the conclusions are biologically meaningful.

Wiener and Poincaré understood this. They articulated a vision of interdisciplinary work grounded in mutual intelligibility: mathematics as a shared language that enables collaboration across expertise boundaries, rather than as a specialized jargon accessible only to initiates. That vision remains highly relevant and has grown more urgent as mathematical methods continue to proliferate in medicine.

Recovering that vision will require deliberate effort. We must change how we train researchers, how we evaluate scientific work, and what we demand from our methods. We must resist the seductive efficiency of black boxes and the false pragmatism that treats interpretability as optional. Most of all, we must refuse to accept the magicalization of mathematics as inevitable—a regrettable yet necessary consequence of complexity and urgency.

It is anything but inevitable. It is a choice we have made, collectively and incrementally, through countless small decisions about what counts as good science. We can make different choices. We can insist that mathematics, however complex, must remain a language—something that can be learned, read, and challenged. We can demand that methods preserve some dimension of transparency: interpretability where possible, rigorous interrogability where full transparency is impossible, and always a commitment to evaluating mechanistic validity rather than accepting correlation as understanding. We can refuse to accept the total collapse of all three dimensions as normal practice.

This will be harder than accepting magic. It will require researchers to invest in skills that publication counts and grant success rarely reward. It will require journals to enforce standards that make papers longer and harder to referee. It will require institutions to support the slow, difficult work of building genuine interdisciplinary expertise.

But the alternative—a biomedical research enterprise built on methods we fail to understand—is untenable. When the reasoning encoded in our methods is not made readable, scrutiny becomes far harder: it grows difficult to distinguish signal from artifact, mechanism from correlation, or insight from overfitting. We are left with a simulacrum of science: empirical validation masquerading as understanding, performance metrics substituting for mechanistic insight.

We can do better. We must do better. The integrity of biomedical research depends on it.
